# Acute Hypersensitivity Reaction After Casirivimab/Imdevimab Infusion in a COVID-19-Positive Young Male: Myopericarditis or Kounis Syndrome?

**DOI:** 10.7759/cureus.31125

**Published:** 2022-11-05

**Authors:** Jose A Rivera, Daniel Aragon, Jose Gomez, Hector Arredondo, Percy M Thomas, Paul Dominici, Olabiyi O Akala, Michael Menowsky

**Affiliations:** 1 Internal Medicine, Doctors Hospital at Renaissance/University of Texas Rio Grande Valley, Edinburg, USA; 2 Emergency Medicine, Doctors Hospital at Renaissance/University of Texas Rio Grande Valley, Edinburg, USA; 3 Emergency Medicine/Critical Care, Doctors Hospital at Renaissance/University of Texas Rio Grande Valley, Edinburg, USA

**Keywords:** unvaccinated, casirivimab and imdevimab, monoclonal antibody infusion, myopericarditis, covid-19

## Abstract

Myocarditis has been a rare, but well-documented side effect of the mRNA-based vaccines against severe acute respiratory syndrome coronavirus 2 (SARS-CoV-2) as well as a complication of viral infections including SARS-CoV-2. However, myopericarditis as a complication of monoclonal antibody infusion or as a complication of allergic reaction to antibody infusions might be underreported.

We report the case of a 30-year-old man with a previous diagnosis of coronavirus disease 2019 (COVID-19) infection one week prior to presentation, unvaccinated for SARS-CoV-2, who was referred from a monoclonal infusion center where he received casirivimab/imdevimab and 15 minutes after the infusion began to complain of chills, chest pain, shortness of breath, and was hypotensive. In the infusion center, the patient received epinephrine and diphenhydramine and was directed to the ER, where the patient was febrile, tachycardic, and hypotensive. Initial troponin was 1.91 which peaked at 11.73 and CK-MB which peaked at 21.2. EKG had no ischemic changes. A two-dimensional echocardiogram showed an ejection fraction (EF) of about 45%, with a left ventricular dysfunction and trivial posterior pericardial effusion, and it was diagnosed as myopericarditis. On admission, he was started on full-dose enoxaparin, aspirin, fluid resuscitation, steroids, remdesevir, and bilevel positive airway pressure (BiPap) due to his respiratory compromise. Three days later, with clinical improvement, a repeat echocardiogram showed EF of 65%, with normal ventricular contractility and no pericardial effusion. The patient was discharged home with close cardiology follow-up.

Though this could be a simple case of viral myopericarditis with troponinemia secondary to demand-ischemia, the differential should be broadened to complication of monoclonal antibody, given the sudden symptom onset after infusion completion and/or a possible Kounis syndrome. Though there have not been any reported cases of casirivimab/imdevimab causing myopericarditis, adverse cardiac events after monoclonal therapy have been reported mainly in cancer patients receiving monoclonal infusions.

## Introduction

Myocarditis has been a rare but well-documented side effect of the mRNA-based vaccines against severe acute respiratory syndrome coronavirus 2 (SARS-CoV-2) as well as a common complication of viral infections including SARS-CoV-2 [1,2]. However, myopericarditis as a complication of monoclonal antibody infusion or as a complication of a hypersensitivity reaction to antibody infusions (Kounis syndrome) might be an underreported adverse reaction [3]. Here, we present a case of a hypersensitivity reaction to monoclonal antibody infusion with a presumptive diagnosis of myopericarditis. 

## Case presentation

A 30-year-old gentleman, unvaccinated for SARS-CoV-2, with a previous diagnosis of coronavirus disease 2019 (COVID-19) infection one week prior to presentation, was referred from a monoclonal infusion center to the ER. Approximately 15 minutes after completing a casirivimab/imdevimab infusion, the patient began to experience chills, chest pain, and shortness of breath and was found to be hypotensive. While in the infusion center, he was treated for suspected hypersensitivity reaction and received epinephrine and diphenhydramine. 

**Figure 1:**
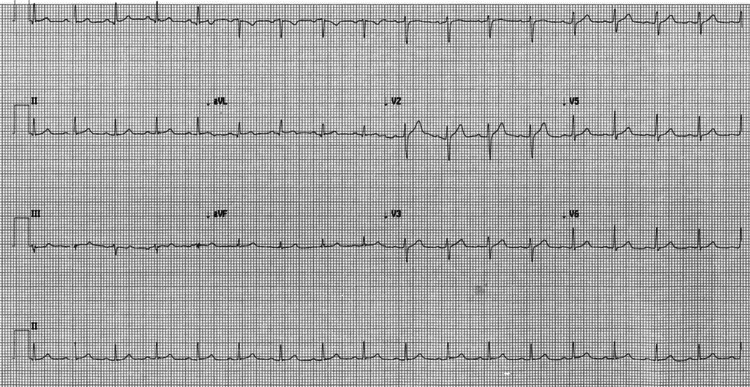
EKG on admission

**Figure 2:**
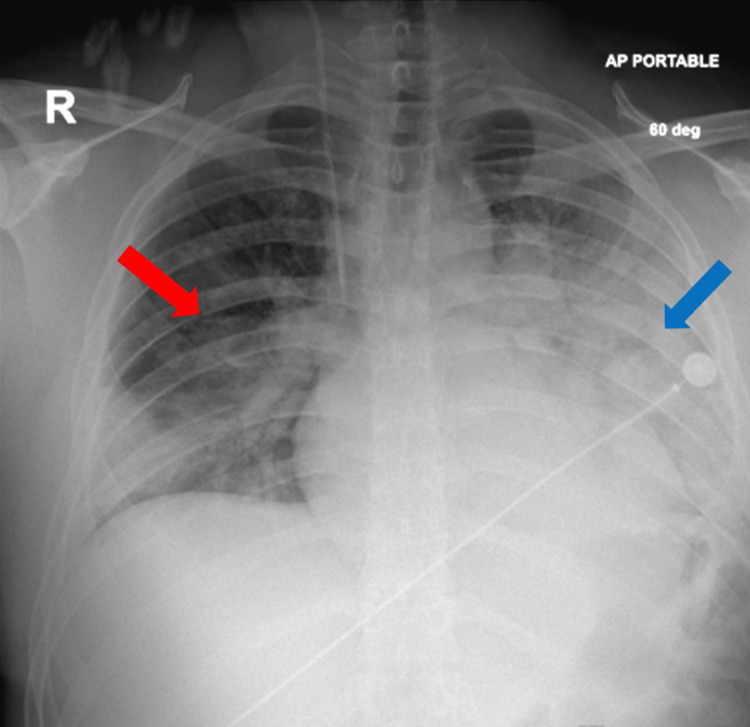
Chest x-ray Multifocal ground-glass infiltrates noted bilaterally (red and blue arrows) greater in the left lung (blue arrow) concerning for COVID-19 pneumonia COVID-19: coronavirus disease 2019

**Figure 3:**
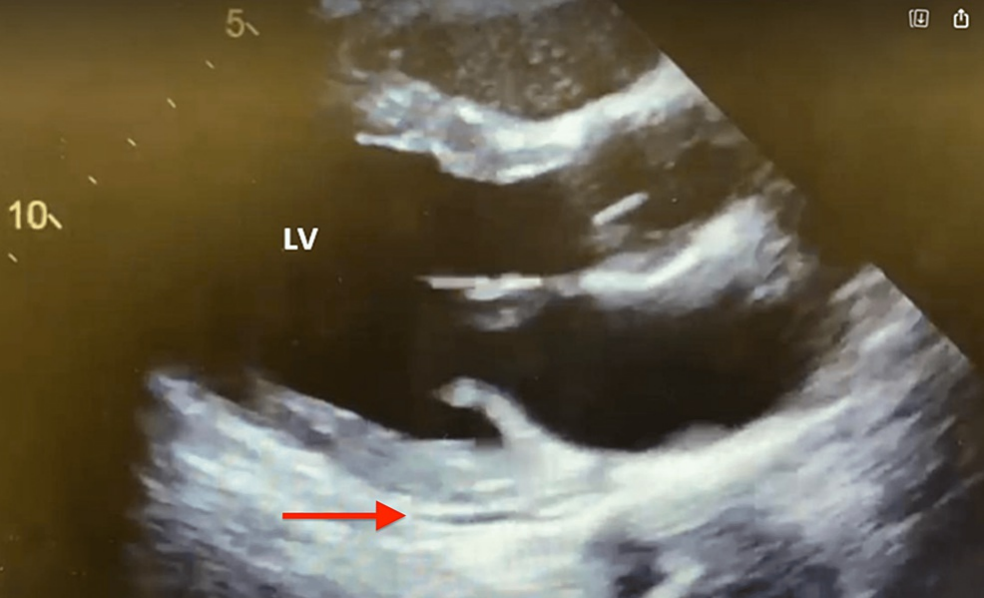
Initial echocardiogram on admission with LV EF 45-50%, mild hypokinesis, and trivial posterior pericardial effusion (red arrow) LV EF: left ventricular ejection fraction

In the ED, the patient was febrile (104.1 ^o^F), tachycardic (109-132 bpm), and hypotensive (mean arterial pressure (MAP) 51-74). On initial presentation, the patient’s oxygen saturation was 99% on room air, but later required noninvasive positive pressure ventilation (NPPV) due to worsening dyspnea. Labs obtained at presentation included an arterial blood gas (ABG) test, which revealed respiratory alkalosis, complete blood count (CBC) and comprehensive metabolic panel (CMP) were grossly unremarkable, and brain natriuretic peptide (BNP), C-reactive protein (CRP), and D-dimer were noted to be elevated (Table 1). Initial troponin was 1.91 ng/ml (normal 0.00-0.04 ng/ml), which peaked at 11.73 ng/ml and began to downtrend on day 2 (Table 2). Creatine kinase-myoglobin binding (CK-MB) peaked at 21.2 ng/ml (normal 30-223 IU/L) and began to downtrend on the same day. Initial EKG had no ischemic changes or significant findings other than sinus tachycardia (Figure 1). Chest X-ray with multifocal ground-glass infiltrates greater in the left lung (Figure 2). An initial two-dimensional echocardiogram done emergently revealed an ejection fraction (EF) of approximately 45%, with left ventricular dysfunction and trivial posterior pericardial effusion (Figure 3). A presumptive diagnosis of myopericarditis was made by the treating cardiologist. 

**Table 1:** Labs on admission BNP: brain natriuretic peptide; CRP: C-reactive protein; SARS-CoV-2: severe acute respiratory syndrome coronavirus 2; PCR: polymerase chain reaction

Test	Results (Normal Values)
BNP	165 pg/mL (0-100 pg/mL)
CRP	28 mg/dL (<1.0 mg/dL)
D-dimer	547 ng/mL (<500 ng/mL)
PCR SARS-CoV-2	Positive

**Table 2:** Troponin level throughout admission

Time	Troponin level (normal 0.00-0.04 ng/ml)
Day 0 18:52	1.91 ng/ml
Day 1 00:50	5.73 ng/ml
Day 1 05:10	11.73 ng/ml
Day 1 12:39	4.70 ng/ml
Day 2 05:19	1.64 ng/ml
Day 3 05:20	0.74 ng/ml

**Figure 4:**
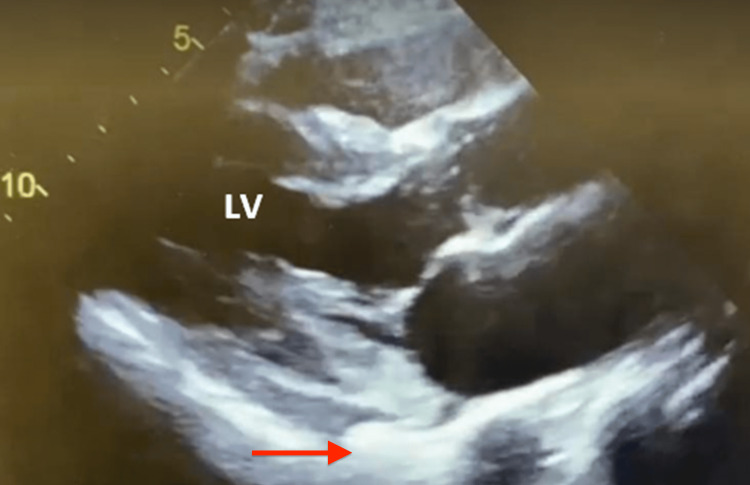
Echocardiogram three days after admission with LV EF 65%, no wall abnormalities, and no pericardial effusion (red arrow) LV EF: left ventricular ejection fraction

The patient was started on full-dose enoxaparin, aspirin, fluid resuscitation, steroids, and remdesivir. Three days later, the patient presented with clinical improvement. Oxygen saturation improved and supplemental oxygen requirements were de-escalated back to room air. A repeat two-dimensional echocardiogram, revealed EF of 65%, with normal ventricular contractility and no pericardial effusion (Figure 4). The patient was discharged home with close cardiology follow-up. 

## Discussion

Myopericarditis is currently defined as the combination of acute pericarditis with coexistent myocarditis usually caused by viral infections, immunopathologic systemic processes, and certain drugs. Characteristics of myopericarditis include findings of elevated troponin levels, diffuse ST elevations on EKG with evidence of myocardial dysfunction, and aspecific findings of increased brightness of the pericardium on two-dimensional echocardiography [4]. Rarely, monoclonal antibody infusions in specific cancer treatments, such as rituximab in hairy cell leukemia, have been found to develop myocarditis.

If we broaden the differential, Kounis syndrome also known as "allergic angina", has been reported as a possible complication of monoclonal infusions secondary to allergic reaction [3,5,6]. Myopericarditis was not found as a possible complication in the landmark study leading to approval for casirivimab/Imdevimab infusion in COVID-19-positive patients; however, reports of allergic reactions are a well-documented adverse event [7].

Since the patient had a positive COVID-19 result in the week prior to presentation and initially did not have severe symptoms requiring hospitalization, he qualified for the administration of casirivimab/imdevimab monoclonal infusion as an outpatient. Minutes after starting infusion therapy, the patient developed systemic symptoms consistent with allergic reactions. Given the landmark study findings, the infusion team responded appropriately by administering epinephrine and diphenhydramine and referring the patient to the ED, where further studies led to the diagnosis of acute myopericarditis.

Even though viral infections are a common cause of myopericarditis, we are unable to clearly differentiate between the infusion or the infection as the cause. The fact that the patient was asymptomatic prior to infusion, developed severe symptoms during the administration, and had rapid resolution of decreased EF and pericardial effusion increases the likelihood that it was caused by the infusion, as viral myopericarditis has a more prolonged resolution (four to six weeks).

## Conclusions

As of this date, there are no reports of documented cases of casirivimab/imdevimab infusion-induced myocarditis. Common side effects of casirivimab/Imdevimab infusion are nausea, vomiting, pruritus, urticaria, chills, and flushing. However, our patient presented with chest pain, shortness of breath, hypotension, elevated troponins, and reduced EF on two-dimensional echocardiogram after the infusion. Therefore, we present a possible case of monoclonal antibody infusion-related myocarditis whether this be as a direct cause or as the allergic reaction caused by the infusion. 
